# An EEG-based study of discrete isometric and isotonic human lower limb muscle contractions

**DOI:** 10.1186/1743-0003-9-35

**Published:** 2012-06-09

**Authors:** Joseph T Gwin, Daniel P Ferris

**Affiliations:** 1 Human Neuromechanics Laboratory, School of Kinesiology, University of Michigan, Ann Arbor, MI, USA

## Abstract

**Background:**

Electroencephalography (EEG) combined with independent component analysis enables functional neuroimaging in dynamic environments including during human locomotion. This type of functional neuroimaging could be a powerful tool for neurological rehabilitation. It could enable clinicians to monitor changes in motor control related cortical dynamics associated with a therapeutic intervention, and it could facilitate noninvasive electrocortical control of devices for assisting limb movement to stimulate activity dependent plasticity. Understanding the relationship between electrocortical dynamics and muscle activity will be helpful for incorporating EEG-based functional neuroimaging into clinical practice. The goal of this study was to use independent component analysis of high-density EEG to test whether we could relate electrocortical dynamics to lower limb muscle activation in a constrained motor task. A secondary goal was to assess the trial-by-trial consistency of the electrocortical dynamics by decoding the type of muscle action.

**Methods:**

We recorded 264-channel EEG while 8 neurologically intact subjects performed isometric and isotonic, knee and ankle exercises at two different effort levels. Adaptive mixture independent component analysis (AMICA) parsed EEG into models of underlying source signals. We generated spectrograms for all electrocortical source signals and used a naïve Bayesian classifier to decode exercise type from trial-by-trial time-frequency data.

**Results:**

AMICA captured different electrocortical source distributions for ankle and knee tasks. The fit of single-trial EEG to these models distinguished knee from ankle tasks with 80% accuracy. Electrocortical spectral modulations in the supplementary motor area were significantly different for isometric and isotonic tasks (p < 0.05). Isometric contractions elicited an event related desynchronization (ERD) in the α-band (8–12 Hz) and β-band (12–30 Hz) at joint torque onset and offset. Isotonic contractions elicited a sustained α- and β-band ERD throughout the trial. Classifiers based on supplementary motor area sources achieved a 4-way classification accuracy of 69% while classifiers based on electrocortical sources in multiple brain regions achieved a 4-way classification accuracy of 87%.

**Conclusions:**

Independent component analysis of EEG reveals unique spatial and spectro-temporal electrocortical properties for different lower limb motor tasks. Using a broad distribution of electrocortical signals may improve classification of human lower limb movements from single-trial EEG.

## Background

Functional neuroimaging could be a powerful tool for neurological rehabilitation. Being able to quantify how task specific brain activation is different in neurologically impaired patients compared to healthy individuals would inform clinical practice, would help clinicians choose a rehabilitation strategy with the best chance of success, and would facilitate tracking of brain plasticity during an intervention [1-3]. In addition, functional neuroimaging may facilitate brain-based control of devices that assist limb movement and thus stimulate activity dependent plasticity [4,5].

Regaining the ability to walk after neurological injury is a fundamental rehabilitation goal that can vastly improve a patient’s lifestyle. This recovery is dependent on our ability to strengthen and modulate cortical inputs for lower limb motor control [2,6]. In addition, the contribution of these cortical inputs, relative to spinal networks, is dependent on task specific body dynamics [7]. To get the most clinical benefit from functional neuroimaging during neurological rehabilitation, it is necessary to establish relationships between electrocortical dynamics and muscle activity in neurologically intact humans during a variety of lower limb motor tasks including individual muscle contractions, coordinated stepping, and locomotion.

Electrical neuroimaging with electroencephalography (EEG) is the only non-invasive brain imaging modality that uses sensors that are light enough to wear while performing dynamic motor tasks and have sufficient time resolution to record changes in brain activity on the timescale of natural human movements [8]. An alternate imaging technique that can be used during dynamic task performance is near-infrared spectroscopy (NIRS). However, NIRS records cortical hemodynamics with a temporal resolution on the order of several seconds [9,10] while EEG records electrocortical processes with a temporal resolution on the order of several ms [11]. Due to these advantages, electrical neuroimaging is well suited for implementation in a clinical rehabilitation setting.

To effectively study electrocortical dynamics using EEG it is necessary to implement signal processing techniques that parse electrocortical contributions to EEG signals from other contributing sources, such as electroocular, electrocardiographic, electromyographic, and movement artifacts. There are many approaches to this problem. Our preferred approach is independent component analysis (ICA). ICA is a blind source separation technique that optimizes a set of maximally independent source signals from linearly mixed recordings. When applied to EEG, ICA parses underlying electrocortical source signals from artifact contaminated electrical potentials on the scalp [12-16]. An advantage of this approach is that electrocortical source signals are analyzed, as opposed to EEG channel signals that reflect the summed contribution of multiple electrocortical sources. In addition, we have recently demonstrated that ICA of EEG allows for functional neuroimaging during human locomotion [17-19]. Therefore, this technique can be used throughout the rehabilitation process as the patient progresses toward more dynamic, real world tasks.

In addition to monitoring cortical plasticity, another potential application of functional neuroimaging for neurological rehabilitation is brain-based control of devices that assist limb movement with the goal of stimulating activity dependent plasticity [4,5]. ICA of EEG may be beneficial for these brain-machine interfaces (BMIs) [20,21]. While early BMIs focused mainly on signals from primary motor cortex, there is an emerging consensus that a broad distribution of signals, and a better understanding of underlying cortical physiology, will improve the information transfer rate in these devices [22]. ICA identifies a broad distribution of electrocortical signals from scalp recordings. In addition, incorporating spatial, spectral, and temporal features of electrocortical signals, across multiple cortical areas, can improve the fidelity of classification algorithms [23-28].

A common approach to the study of electrocortical source signals is to evaluate modulations in spectral power that are time-locked to an event of interest. One well established phenomenon is that oscillatory cortical activity in the α-band (8–12 Hz) and β-band (12–30 Hz) is suppressed during dynamic movements [29-31]. This phenomenon is referred to as event-related desynchronization (ERD) and has been studied extensively for upper limb movements and to a lesser degree for foot and toe movements [32-34]. Most studies evaluate ERD in EEG channel signals. Electrocorticography provides a more direct measure of the underlying electrocortical sources, but electrocorticography is also affected by volume conduction of multiple electrocortical source signals [35]. ICA provides a means to evaluate spectral modulations in the underlying electrocortical processes themselves. In one study, ICA of EEG was shown to enhance the ERD associated with finger movements [36].

In this study, we used ICA of high-density EEG to examine electrocortical dynamics while 8 healthy subjects performed isometric and isotonic, knee and ankle, flexor and extensor muscle contractions at two different effort levels. The goals of this study were to characterize differences in spatial and spectro-temporal electrocortical dynamics associated with these muscle activations, as well as to assess the trial-by-trial (i.e., single exercise repetition) consistency of these differences by decoding the type of muscle activation from the recorded brain signals. Specifically, we tested 1) whether the fit of single-trial EEG to different ICA mixture models could distinguish knee from ankle contractions; 2) if muscle contraction related electrocortical spectral modulations in the motor cortex would differ between isometric and isotonic tasks, and between flexion and extension tasks; 3) if tasks requiring a greater muscular effort would elicit a more pronounced ERD; and 4) if muscle contraction type could be distinguished from single-trial electrocortical spectrograms.

Studying these electrocortical dynamics will provide a better understanding of lower limb motor control and may inform our interpretation of earlier results regarding electrocortical spectral modulations during human walking. The techniques that we have implemented in this study can be used throughout the rehabilitation process to study both discrete lower limb muscle activations and more dynamic tasks, such as coordinated non-weight-bearing stepping or normal locomotion. Therefore, we believe that the results of this study may have implications for neurorehabilitation of gait, including monitoring cortical plasticity and providing real-time control of robotic lower limb exoskeletons.

## Methods

### Tasks

Eight healthy volunteers with no history of major lower limb injury and no known neurological or musculoskeletal deficits completed this study (7 males; 1 female; age range 21–31 years). Subjects provided written informed consent prior to the experiment. The University of Michigan Internal Review Board approved all procedures, which complied with the standards defined in the Declaration of Helsinki.

**Figure 1:**
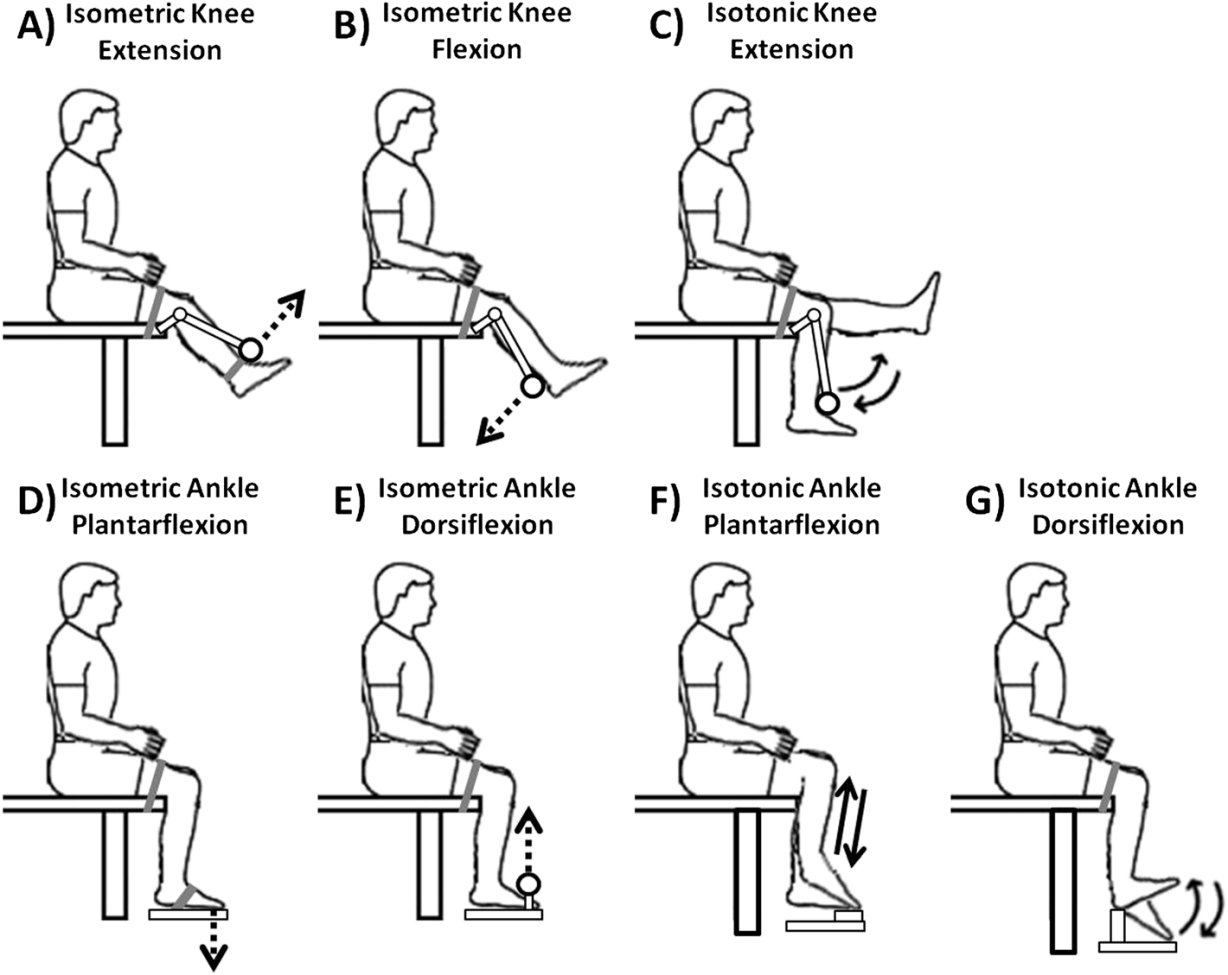
**A sketch of the experimental setup for A) isometric knee extension, B) isometric knee flexion, C) isotonic knee extension, D) isometric ankle plantar flexion, E) isometric ankle dorsiflexion, F) isotonic ankle plantar flexion, and G) isotonic ankle dorsiflexion.** For isometric exercises the direction of the applied force is indicated by a dashed arrow. For isotonic exercises the direction of movement is indicated by solid arrows.

Subjects sat on a bench while performing isometric muscle activations (activation without limb movement) and isotonic movements (activation with limb movement, concentric followed by eccentric) of the knee and ankle joints (Figure 1). Subjects performed both flexion and extension; except for isotonic knee flexion, which could not be accommodated by the test apparatus. They completed two sets of 20 repetitions of each exercise. One set was performed at high effort and the second set was performed at low effort. For high effort isotonic exercises, we applied the following weights: 9.1 kg (20 lbs) on top of the knee for plantar flexion; 3.2 kg (7 lbs) on the dorsal surface of the foot for dorsiflexion; 9.1 kg (20 lbs) on the anterior shank just proximal to the ankle for knee extension. For low effort isotonic exercises, we did not apply weight (movement was inhibited only by the mass of the limbs). For high effort isometric exercises, we instructed subjects to “press as hard as you can using only your leg, keep your arms and torso still, and don’t grab the exercise bench with your hands.” For low effort isometric exercises, we instructed subjects to “push approximately 25% as hard as you did for the high effort set.” Subjects were not given visual feedback of the force or torque they exerted. A few practice repetitions allowed subjects to acclimate to 25% effort. Isometric and isotonic exercise repetitions were performed over roughly 3 seconds. For isotonic tasks the concentric and eccentric contractions were performed continuously (i.e., immediate direction change after the concentric contraction) and took a total of 3 seconds. Subjects paused for 5 seconds between repetitions. We did not provide timing cues because we did not want to confound electrocortical dynamics with an audio or visual task. As a result, exercise timing was approximate. Subjects performed isometric ankle exercises at a neutral ankle angle and isometric knee exercises at 45 degrees of flexion. All exercises were performed with the right lower limb only. We screened subjects for handedness, by asking them their preferred writing hand, and footedness, by asking which foot they would kick a ball with. All were right handed and right footed.

### Recording EEG and lower limb dynamics

We recorded EEG at 512 Hz using an ActiveTwo amplifier and a 264-channel active electrode array (BioSemi, Amsterdam, The Netherlands). A digitizer (Polhemus, Colchester, VT, USA) localized the 256-channel EEG head cap, as well as 8 electrodes that were external to the head cap, with respect to anatomic head reference points (nasion, left preauricular point, and right preauricular point). After data collection, we applied a zero phase lag 1 Hz high-pass Butterworth filter to the EEG signals to remove drift. We removed EEG signals exhibiting substantial noise throughout the collection in a manner similar to [17,18]. Channels with standard deviation ≥ 1000 μV were removed. Any channel whose kurtosis was more than 3 standard deviations from the mean was removed. Channels that were uncorrelated (r ≤ 0.4) with nearby channels for more than 0.1% of the time-samples were removed. On average, 191 channels were retained (range: 134–240; standard deviation: 34.6). The remaining channels were evenly distributed around the head; the mean (standard deviation) channel rejection rate was 72.4% (20.2%). The remaining channels were re-referenced to an average reference. We performed all processing and analysis in Matlab (The Mathworks, Natick, MA) using scripts based on EEGLAB [37], an open source Matlab toolbox for processing electrophysiological data. For isotonic exercises, we measured ankle and knee angles using electrogoniometers (Biometrics, Gwent, England). For isometric exercises, we measured force production using a load cell (Omegadyne, Sunbury, OH, USA). We sampled the load cell and electrogoniometers at 1000 Hz, and synchronized EEG and biomechanics signals offline.

### Adaptive mixture independent component analysis

For each subject, we merged EEG signals from all conditions into a single dataset. We submitted these data to an adaptive mixture ICA algorithm [AMICA] [38,39], which generalizes infomax [40,41] and multiple mixture [42,43] ICA approaches. AMICA is an open source plugin for EEGLAB (sccn.ucsd.edu/eeglab/plugins.html) that generates a predetermined number of mixture models each of which captures a competition selected subset of the data. Based on the known somatotopic distribution of the sensorimotor cortex [44] we had an *a priori* hypothesis that ankle and knee muscle actions would elicit different electrocortical source spatial distributions. Therefore, we allowed AMICA to generate 2 mixture models. For each subject, we separately computed model probabilities for the subset of data containing all ankle trials and the subset of data containing all knee trials. Model probability reflects the likelihood that a given model best fits a particular subset of data (on a scale of 0 to 1) and was computed based on the posterior log-likelihood using Matlab functions in the AMICA plugin for EEGLAB. Analysis of variance assessed whether the model probabilities were significantly different across all subjects.

DIPFIT functions within EEGLAB computed an equivalent current dipole model that best explained the scalp topography of each independent component using a boundary element head model based on the Montreal Neurological Institute (MNI) template (the average of 152 MRI scans from healthy subjects, available at http://www.mni.mcgill.ca) [45]. We aligned digitized electrode locations with the head model by scaling and rotating the head coordinate system so that the digitized anatomical reference points matched the head model anatomic reference points. We excluded independent components if the projection of the equivalent current dipole to the scalp accounted for less than 85% of the scalp map variance, or if the topography, time-course, and spectra of the independent component were reflective of eye movement or electromyographic artifact [13,14]. The remaining independent components reflected electrocortical sources. These sources were clustered across subjects using EEGLAB routines that implemented k-means clustering on vectors coding differences in equivalent dipole locations and the topography of the dipole projection to the scalp. Scalp topography was reduced to 10 principal dimensions using principal component analysis. To account for differences in the dimensions of the dipole locations compared to the scalp topography, we gave dipole locations a weight of 3 and topography principle components a weight of 1 prior to clustering, as in [17,18]. We retained clusters that contained electrocortical sources from at least 6 of 8 subjects; electrocortical sources that were not included in these clusters were excluded from all further analyses.

### Electrocortical source time-frequency analysis

To test the hypothesis that different types of muscle activations have different electrocortical spectro-temporal features, we generated spectrograms for each electrocortical source, each muscle contraction, and each subject. We performed time-frequency analysis using Morlet wavelets with 500 ms sliding windows and 25 ms of overlap. Frequencies were divided into 220 log spaced bins from 3 to 150 Hz. We time-locked single-trial spectrograms to the start of each trial and then linearly time-warped them so that the end of the trial occurred at the same adjusted latency in each spectrogram. We determined the start and end of isometric trials based on the onset and offset of applied force (load cell measurements). We determined the start and end of isotonic trials based on the onset and offset of joint rotation (electrogoniometer measurements). We normalized each spectrogram by subtracting the average log spectrum for a pre-trial baseline (1000 ms to 500 ms prior to onset) from the spectrogram (this is a static baseline, each exercise repetition was preceded by a 5 second pause). We then generated grand average normalized spectrograms in the α- and β-bands for electrocortical sources in the contralateral medial sensorimotor cortex for flexion, extension, isometric, and isotonic trials. We performed pairwise comparisons of these spectrograms using a bootstrapping method available within EEGLAB [37]. Finding no significant differences in these spectrograms for knee and ankle muscle trials or for flexion and extension trials, we averaged the spectrograms across these conditions yielding distinct grand average spectrograms for the following four conditions: isometric low effort, isometric high effort, isotonic low effort, and isotonic high effort. Significant fluctuations from baseline in these grand average spectrograms were identified using EEGLAB bootstrapping methods [37]. Last, within contraction type (i.e., isometric or isotonic) T-tests compared the means of the α- and β-band time-frequency points that exhibited a significant spectral change from baseline for low effort versus high effort trials.

### 4-way classification of single trial electrocortical source spectrograms

We evaluated two 4-way linear naïve Bayesian classifiers for grouping single trial data as isometric or isotonic and high or low effort. The first classifier was based only on the cluster of electrocortical sources in the supplementary motor area and the second classifier was based on all electrocortical sources except for those in the visual cortex. The second classifier was included to evaluate the extent to which the addition of electrocortical sources that were not in the supplementary motor area would improve the fidelity of the classification algorithm. For this classifier, electrocortical sources in the visual cortex were excluded for control purposes. Subjects were instructed not to look at the lower limb during testing but differences in eye gaze between conditions could have biased electrocortical dynamics in the visual cortex.

For both classifiers, feature vectors were generated by reducing the resolution of the normalized spectrograms by a factor of 10 (in both time and frequency) and identifying significant time-frequency points from the reduced resolution spectrograms across trials for each subject and each type of muscle activation. The decibel values at the time-frequency points that were significant across trials were selected in each trial and formed the single-trial feature vector. Next, we trained and tested subject specific linear naïve Bayesian classifiers (i.e., classifiers were trained and tested on single subject data) using the Matlab Statistics Toolbox. For each subject, a 10-fold cross validation was performed. The confusion matrices for each subject and each fold were then averaged to form a grand average confusion matrix for each classifier.

## Results

**Figure 2:**
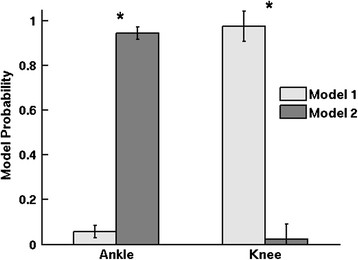
**AMICA model probabilities for ankle trials (left) and knee trials (right).** Error bars show 1 SD. * p < 0.01.

The differences between the model probabilities for the 2-model AMICA decomposition were significant for the subset of data for knee tasks and the subset of data for ankle tasks (p < 0.01). In other words, one model best explained the data during knee exercises and the other model best explained the data during ankle exercises (Figure 2). For clarity, these AMICA models are referred to as the knee model and the ankle model, respectively, for the remainder of this manuscript. However, it is critical to recall that these models were trained on the entire dataset without knowledge of the underlying muscles being activated. On a trial-by-trial basis, the fit of the recorded EEG to the AMICA mixture models distinguished knee contractions from ankle contractions with 80% accuracy.

**Figure 3:**
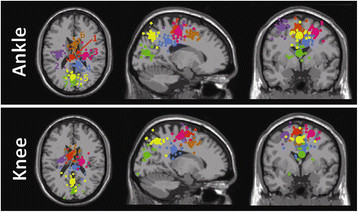
**Clusters of electrocortical source equivalent current dipoles localized to the (1: orange) supplementary motor area, (2: purple) left dorsal premotor area, (3: magenta) right dorsal premotor area, (4: blue) posterior cingulate, (5: yellow) posterior parietal, (6: brown) anterior cingulate, and (7: green) visual cortex.** Two dipole models are shown; (top) the model best fitting the EEG signals during ankle exercises and (bottom) knee exercises. Small spheres indicate dipole locations for single electrocortical sources for single subjects; larger spheres indicate geometric cluster centroids

The knee and ankle ICA mixture models parsed an average of 23.8 and 21.8 electrocortical sources from the EEG signals, respectively. The number of electrocortical sources per subject was not significantly different between the two models (ANOVA, p = 0.66). Clusters containing electrocortical sources from at least 6 of 8 subjects were localized to the anterior cingulate, posterior cingulate, supplementary motor, left dorsal premotor right dorsal premotor, posterior parietal, and visual cortex. All of these clusters were present in both the knee and ankle ICA models (Figure 3). Talairach coordinates for the cluster centroids are shown in Table 1.

**Table 1:** Talairach coordinates for the geometric cluster centroids

		** Knee Model**		** Ankle Model**	
Cluster	Nearest Grey Matter	Talairach	Distance to	Talairach	Distance to
			Nearest Grey		Nearest Grey
	(Brodmann Area) ^1^	Coordinates	Matter (mm)^1^	Coordinates	Matter (mm)^1^
1: supplementary motor area	BA 6	(−6, -2, 58)	1	(−4, -21, 55)	0
2: left dorsal premotor area	BA 6	(−25,-10,53)	0	(−31, -14, 55)	2
3: right dorsal premotor area	BA 6	(20, -13, 55)	3	(26, -16, 47)	5
4: posterior cingulate	BA 23, 31	(5, -26, 28)	0	(10, -36, 31)	3
5: posterior parietal	BA 7	(−1, -58, 44)	0	(−2, -65, 41)	1
6: anterior cingulate	BA 24, 32	(2, 6, 40)	2	(2, 7, 43)	4
7: visual	BA 18	(3, -77, 18)	0	(−2, -74, 10)	0

^1^ Determined using the freely downloadable Talairach Client (http://www.talairach.org) [46]

The cluster numbers correspond to the numeric labels in Figure 3

**Figure 4:**
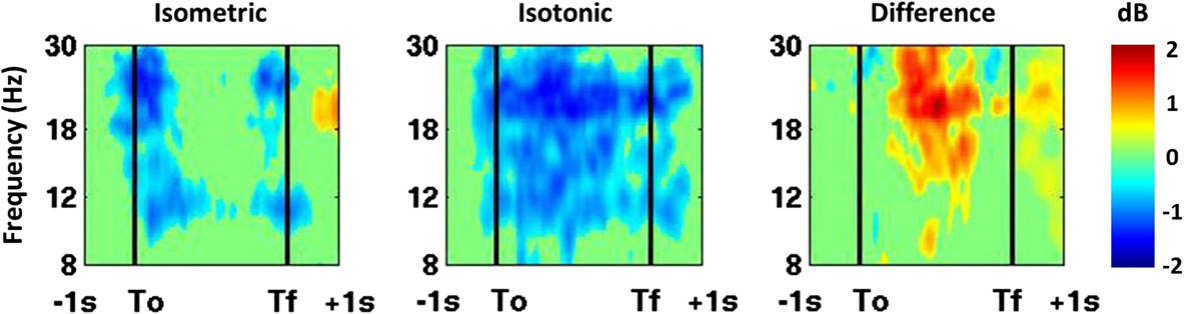
**Grand average normalized spectrograms for supplementary motor area electrocortical sources showing average changes in spectral power during the task relative to a pre-trial baseline for isometric (left) versus isotonic (middle) trials.** The right panel shows the difference between isometric and isotonic conditions. The horizontal axis begins 1 s prior to trial onset (T_o_; first black vertical line) and ends 1 s after trial offset (T_f_; second black vertical line). The times between the onset and offset of the trials were warped to align these latencies across all trials. Non-significant changes from baseline (p > 0.05) were set to 0 dB (green).

**Figure 5:**
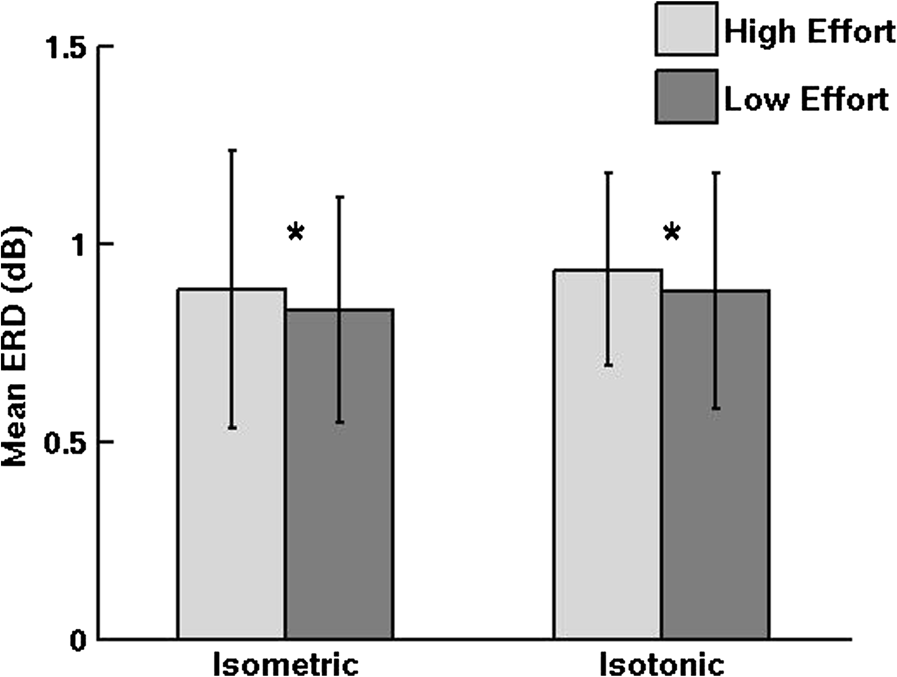
**Average event-related desyncronization (ERD) for high effort and low effort muscle contractions shown separately for isometric (left) and isotonic (right) conditions.** Error bars show 1 SD. * p < 0.01.

Isometric and isotonic contractions elicited significantly different α- and β-band spectral power modulations for the cluster of electrocortical sources in the supplementary motor area (cluster 1: orange in Figure 3). Specifically, isometric contractions elicited α- and β-band ERD at trial onset and offset while isotonic contractions elicited a sustained α- and β-band ERD throughout the trial (Figure 4). Finding no significant differences in these spectrograms for knee and ankle muscle trials or for flexion and extension trials, we averaged the spectrograms across these conditions. For both isometric and isotonic contractions, high effort tasks elicited a slightly but significantly (p < 0.01, power > 0.99) more pronounced ERD (Figure 5).

**Figure 6:**
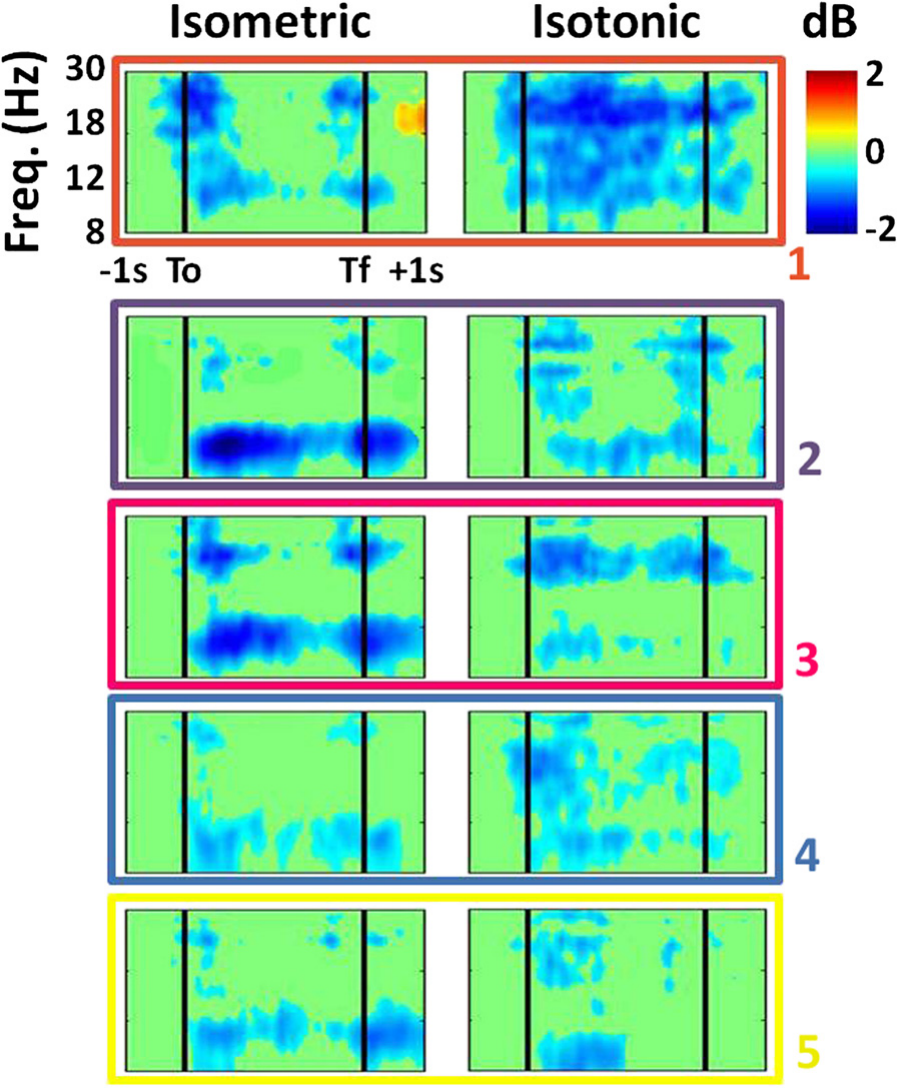
**Grand average normalized spectrograms for (top row) supplementary motor area, (second row) left dorsal premotor area, (third row) right dorsal premotor area, (fourth row) posterior cingulate, and (fifth row) posterior parietal cortex showing average changes in spectral power during the task relative to a −1000 ms to −500 ms baseline for isometric (left) and isotonic (right) trials.** The color of the border and the numeric label for each row corresponds to the color and numeric label of the dipoles for the corresponding cluster shown in Figure 3. The horizontal axis begins 1 s prior to trial onset (T_o_; first black vertical line) and ends 1 s after trial offset (T_f_; second black vertical line). The times between the onset and offset of the trials were warped to align these latencies across all trials. Non-significant changes from baseline (p > 0.05) were set to 0 dB (green).

We evaluated two 4-way linear naïve Bayesian classifiers for grouping single trial data as isometric or isotonic and high or low effort. Finding no significant differences in the spectrograms for flexion and extension we did not attempt to decode these conditions. The first classifier was based only on electrocortical sources in the supplementary motor area (cluster 1: orange in Figure 3) and the second classifier was based on all electrocortical sources except for those in the visual cortex. Grand average spectrograms for each cluster of electrocortical sources used in the second classifier are shown in Figure 6. Spectrograms for the anterior cingulate cortex are excluded because no significant differences from baseline were found for this cluster. The accuracies of these classifiers were 68.8 ± 9.3% and 87.1 ± 9.0% (mean ± SD), respectively. The grand average normalized confusion matrices, averaged across 10 folds and 8 subjects, are shown in Tables 2 and 3, respectively.

**Table 2:** Grand average normalized confusion matrix for the 4-way linear naïve Bayesian classifier using electrocortical sources in the supplementary motor area

**Actual**	**Predicted**			
	**High Effort**	**Low Effort**	**High Effort**	**Low Effort**
	**Isometric**	**Isometric**	**Isotonic**	**Isotonic**
High Effort Isometric	20.0%	4.7%	2.8%	3.0%
Low Effort Isometric	4.4%	19.4%	2.7%	2.9%
High Effort Isotonic	2.3%	2.4%	15.9%	2.2%
Low Effort Isotonic	1.1%	1.0%	1.4%	13.8%

**Table 3:** Grand average normalized confusion matrix for the 4-way linear naïve Bayesian classifier using all electrocortical sources except those in the visual cortex

**Actual**	**Predicted**			
	**High Effort**	**Low Effort**	**High Effort**	**Low Effort**
	**Isometric**	**Isometric**	**Isotonic**	**Isotonic**
High Effort Isometric	25.8%	2.7%	1.3%	1.7%
Low Effort Isometric	2.1%	24.8%	1.0%	1.0%
High Effort Isotonic	0.5%	0.5%	19.0%	1.0%
Low Effort Isotonic	0.2%	0.3%	0.9%	17.3%

## Discussion

We used high-density EEG to study voluntary lower limb isometric and isotonic, ankle and knee, flexor and extensor muscle contractions in eight healthy subjects. The goals of this study were to characterize differences in electrocortical dynamics between these muscle actions and to assess the trial-by-trial consistency of these differences by decoding the type of muscle action from recorded brain signals.

For all subjects, AMICA captured different spatial distributions of electrocortical sources for ankle and knee actions. The somatotopic arrangement of the sensory and motor cortices is well established [44]. Therefore, from a physiological perspective it is not surprising that knee and ankle muscle actions would elicit different distributions of underlying electrocortical sources. The supplementary motor area (cluster 1: orange in Figure 3) is of particular interest because this region of the premotor cortex projects to distal limb motor nuclei while the dorsal premotor area (clusters 2 and 3: purple and magenta in Figure 3) projects mainly to motor nuclei innervating the proximal limb musculature [47]. The location of face, hand, and foot areas of the human supplementary motor area follow an anterior-posterior shift [48]. Therefore, the posterior shift of the supplementary motor area ankle cluster compared to the knee cluster is consistent with expected somatotopy; though supplementary motor area somatotopy has not been formalized to the extent that primary motor cortex somatotopy has been. However, we do not believe that the data collected here provides a sufficient basis for a physiological explanation for the subtle location shift of this cluster between the two models. Future work should evaluate the use of subject specific head models (derived from individual magnetic resonance images) to improve the accuracy of source localization. Nevertheless, AMICA provides a novel data driven way to derive distinct source distributions. In this study, we could have separated the ankle and knee data *a priori* and submitted these data to two distinct ICA decompositions. The benefit of AMICA is that *a priori* knowledge of different source distributions is not required. For this reason we choose to evaluate AMICA in this study. Our results suggest that AMICA of high-density EEG has sufficient spatial resolution to distinguish electrocortical process for knee tasks from those for ankle tasks.

We used the fit of single-trial EEG to the AMICA mixture models to distinguished knee from ankle tasks with modest success. Isometric knee exercises could be distinguished from isometric ankle exercises with 91% accuracy, but isotonic exercises could only be distinguished with 62% accuracy. For some subjects (3 of 8), 100% accuracy was achieved for both isometric and isotonic exercises. For other subjects, isometric exercises were accurately categorized but isotonic exercises were not. We expect that for these subjects, the isotonic exercises elicited a distribution of electrocortical activity that was different from that elicited by the isometric exercises. It might be helpful for future studies to allow the AMICA algorithm to identify additional mixture models, but this might require larger data sets. In fact, this observation highlights an important benefit of the AMICA algorithm

Spectrograms for electrocortical sources in the supplementary motor area differed between isometric and isotonic contractions, but did not differ significantly between flexion and extension trials. Specifically, isometric contractions elicited an ERD in the α- and β-band at force onset and offset while isotonic contractions elicited a sustained α- and β-band ERD throughout the trial. In addition, high effort trials (i.e., greater muscle activation) elicited a slightly but significantly more pronounced desynchronization than low effort trials. This result is consistent with the understanding that oscillatory cortical activity in the α- and β-bands reflects steady-state sensorimotor processing that is reduced during dynamic movement [29-31]. Regarding the ERD during isometric contractions at onset and offset, it is important to note that the onset of an isometric contraction consists of dynamic muscle shortening and tendon lengthening until the desired level of force is achieved, and the offset of an isometric contraction consists of muscle lengthening and tendon shortening until rest is achieved [49]. In addition, some limb movement is inevitable as the test apparatus and the soft tissues of the lower limb becomes loaded and then unload at the onset and offset of each trial. To our knowledge this is the first comparison of electrocortical dynamics associated with isometric and isotonic lower limb muscle activations.

Several observations can be made from the electrocortical cluster spectrograms shown in Figure 6. First, while the α-band ERD for isometric trials occurred only at trial onset and offset for the supplementary motor area cluster (first row of Figure 6), the α-band ERD was persistent throughout the isometric trials for the dorsal premotor area clusters that were located more laterally in the premotor cortex (second and third row of Figure 6, respectively). This may be the result of dynamic torso stabilization throughout the trial. Second, significant ERD for the supplementary motor area cluster preceded trial onset by roughly 400 ms but significant ERD for all other electrocortical source clusters did not begin until after trial onset. Third, the supplementary motor area ERD was β-dominant for both the isometric and isotonic conditions; whereas the dorsal premotor area clusters were α-dominant for the isometric condition and β-dominant for the isotonic conditions. Most importantly, electrocortical spectrograms in broadly distributed brain regions contained information regarding the level of effort and the contraction type (isometric versus isotonic). This is evidenced by the fact that classifiers based only on supplementary motor area electrocortical sources achieved a 4-way classification accuracy of 69% while classifiers based on electrocortical sources in multiple brain regions achieved a 4-way classification accuracy of 87%. This findings supports the notion that a broad distribution of electrocortical signals will improve the information transfer rate in BMIs [22].

In this study we used ICA to parse EEG signals recorded on the scalp into underlying electrocortical source signals and then evaluated the spectro-temporal characteristics of the source signals. However, this is certainly not the only way to study electrocortical dynamics associated with limb movements. Many informative findings have come from time-domain measures, such as motor-related cortical potentials (MRCPs). MRCPs have been shown to be greater and occur earlier for eccentric elbow contractions than for concentric contractions [50]. In addition, MRCP amplitude has been shown to scale with the amount of torque produced [51] and to decrease with fatigue [52]. In addition, coherence analysis of EEG and EMG could provide additional insight. In particular, such analysis could be used to localize active brain regions for knee and ankle tasks.

There are certain limitations of this study. First, it is possible that certain sources of electromyographic or electroocular artifact were present during ankle and not knee trials (or vice versa), even after removing artifacts using ICA. This would have positively influenced the prediction of knee vs. ankle action. To avoid this, subjects were seated in the same position for all experimental conditions and were instructed to keep their gaze forward. In addition, they were instructed to engage only the right leg during each exercise (i.e., subjects were not permitted to grab the exercise bench to generate more force). Second, the exercises that were categorized as isotonic in this study are not truly isotonic (joint torque was not constant throughout the trial), and this could confound the interpretation of the data. Third, the methods of classification used in this study cannot be used for real-time classification. ICA mixture models were trained offline. It remains to be seen whether ICA mixture models are stable from day-to-day, in the presence of unavoidable differences in EEG head-cap setup. If the mixture models are stable from day-to-day then subject specific mixture models could be applied in real-time. In addition, classification of muscle contraction type was based on time-frequency data for the full (approximately 3 second) repetition. Real-time classification would require the use of a shorter duration of EEG activity. However, the purpose of decoding single-trial spectrograms in this study was to assess the trial-by-trial consistency of the task specific differences in the electrocortical spectrograms.

The results of this study demonstrate that ICA of high-density EEG can be used to monitor a broad distribution of electrocortical sources that contribute to lower limb muscle actions. In an earlier study we used a similar imaging technique during human locomotion and found spectral modulations in sensorimotor, anterior cingulate, and posterior parietal cortex that were locked to the gait cycle [17]. It remains to be seen how these task specific electrocortical dynamics are affected by neurological injuries, such as stroke or spinal cord injury, or how they change in response to motor rehabilitation. However, alternative imaging techniques suggest that functional recovery will rely on plasticity in multiple cortical regions and that the relative contribution of different regions will change throughout the course of rehabilitation [6,53-57]. The techniques used in this study may provide a means to better understand the cortical physiology underlying neurological rehabilitation and recovery.

## Conclusions

We used high-density EEG to study electrocortical dynamics in healthy subjects performing isometric and isotonic, knee and ankle, flexor and extensor muscle contractions on a dynamometer. AMICA parsed EEG recordings into two different models of underlying source signals, one of which best explained the variance in the EEG recorded during knee exercises and the other during ankle exercises. This suggests that AMICA of high-density EEG has sufficient spatial resolution to distinguish electrocortical processes for knee tasks from those for ankle tasks. In fact, we found that the fit of single-trial EEG to the AMICA models distinguished knee from ankle actions with 80% accuracy.

We also examined electrocortical spectral modulations during the tasks and found that isometric contractions elicited α- and β-band ERD in the supplementary motor area at trial onset and offset while isotonic contractions elicited a sustained α- and β-band ERD throughout the trial. We found that classifiers based on electrocortical sources in the supplementary motor area could classify single trial spectrograms into one of four groups (high effort isometric, low effort isometric, high effort isotonic, and low effort isotonic) with 69% accuracy while classifiers based on electrocortical sources in multiple brain regions achieved a 4-way classification accuracy of 87%.

Our results demonstrate that different types of lower limb muscle activation carry unique spatial and spectro-temporal electrocortical signatures, and that a broad distribution of electrocortical signals may improve classification of human lower limb movements from single-trial EEG data. Our findings may have implications for tracking cortical plasticity during neurorehabilitation. Specifically, the techniques presented here could be used to track changes in spectro-temporal and spatial properties of motor-related electrocortical signals during recovery. This could help researchers and clinicians gauge the success of a therapy or pharmaceutical treatment.

## Abbreviations

AMICA: adaptive mixture independent component analysis; BMI: brain machine interface; EEG: electroencephalography; ERD: event-related desynchronization; ICA: independent component analysis.

## Competing interests

The authors declare that they have no competing interests.

## Authors’ contributions

JG and DF collectively conceived and designed the study. JG collected and analyzed data and drafted the manuscript. JG and DF read and approved the final manuscript.
